# Genetic load and transgenic mitigating genes in transgenic *Brassica rapa *(field mustard) × *Brassica napus *(oilseed rape) hybrid populations

**DOI:** 10.1186/1472-6750-9-93

**Published:** 2009-10-31

**Authors:** Christy W Rose, Reginald J Millwood, Hong S Moon, Murali R Rao, Matthew D Halfhill, Paul L Raymer, Suzanne I Warwick, Hani Al-Ahmad, Jonathan Gressel, C Neal Stewart

**Affiliations:** 1 Department of Plant Sciences, University of Tennessee, Knoxville, TN 37966 USA; 2 Department of Biology, St. Ambrose University, Davenport, IA 52803 USA; 3 Department of Crop and Soil Sciences, University of Georgia, 1109 Experiment Street, Georgia Station, Griffin, GA 30223 USA; 4 Agriculture and Agri-food Canada, Eastern Cereal and Oilseeds Research Centre, Ottawa, Ontario K1A 0C6, Canada; 5 Department of Biology & Biotechnology, An-Najah National University Nablus, Palestine; 6 Department of Plant Sciences, Weizmann Institute of Science, Rehovot 76100, Israel

## Abstract

**Background:**

One theoretical explanation for the relatively poor performance of *Brassica rapa *(weed) × *Brassica napus *(crop) transgenic hybrids suggests that hybridization imparts a negative genetic load. Consequently, in hybrids genetic load could overshadow any benefits of fitness enhancing transgenes and become the limiting factor in transgenic hybrid persistence. Two types of genetic load were analyzed in this study: random/linkage-derived genetic load, and directly incorporated genetic load using a transgenic mitigation (TM) strategy. In order to measure the effects of random genetic load, hybrid productivity (seed yield and biomass) was correlated with crop- and weed-specific AFLP genomic markers. This portion of the study was designed to answer whether or not weed × transgenic crop hybrids possessing more crop genes were less competitive than hybrids containing fewer crop genes. The effects of directly incorporated genetic load (TM) were analyzed through transgene persistence data. TM strategies are proposed to decrease transgene persistence if gene flow and subsequent transgene introgression to a wild host were to occur.

**Results:**

In the absence of interspecific competition, transgenic weed × crop hybrids benefited from having more crop-specific alleles. There was a positive correlation between performance and number of *B. napus *crop-specific AFLP markers [seed yield vs. marker number (r = 0.54, P = 0.0003) and vegetative dry biomass vs. marker number (r = 0.44, P = 0.005)]. However under interspecific competition with wheat or more weed-like conditions (i.e. representing a situation where hybrid plants emerge as volunteer weeds in subsequent cropping systems), there was a positive correlation between the number of *B. rapa *weed-specific AFLP markers and seed yield (r = 0.70, P = 0.0001), although no such correlation was detected for vegetative biomass. When genetic load was directly incorporated into the hybrid genome, by inserting a fitness-mitigating dwarfing gene that that is beneficial for crops but deleterious for weeds (a transgene mitigation measure), there was a dramatic decrease in the number of transgenic hybrid progeny persisting in the population.

**Conclusion:**

The effects of genetic load of crop and in some situations, weed alleles might be beneficial under certain environmental conditions. However, when genetic load was directly incorporated into transgenic events, e.g., using a TM construct, the number of transgenic hybrids and persistence in weedy genomic backgrounds was significantly decreased.

## Background

Over the past dozen years, a number of crops, such as soybean, maize, rice, cotton and canola, have been genetically engineered to contain a variety of fitness enhancing transgenes. Some of these transgenes can increase a crop's defenses by conferring resistance to a number of diseases, herbicides, abiotic stresses, and yield reducing herbivores [1]. Consequently, there are environmental and regulatory concerns about the adventitious presence of transgenes, especially with regards to hybridization and introgression into weedy relatives [2]. Specifically, could the introgression of fitness-enhancing transgenes result in new hard-to-manage, weedy or invasive biotypes possessing competitive advantages such as herbicide resistance, drought and salt tolerance, or pathogen and insect resistance? If this were to occur, these hybrids could potentially disrupt agricultural and non-agricultural systems [2-16].

Transgene introgression and competition has been studied in many weed × crop systems including *Brassica rapa *(field mustard) × *Brassica napus *(canola). Halfhill et al. (2005) analyzed the fitness of four *B. rapa *× transgenic *B. napus *backcrossed transgenic hybrid lines that originated from a single transgenic event. They found that the average vegetative growth and nitrogen use efficiency of the transgenic hybrids were lower than the wild-type *B. rapa *parent indicating that the transgenic *Brassica *hybrids would likely be less fit in an agronomic setting. They concluded that the observed decrease in fitness could be the result of species or hybridization effects, initial transgene insertion loci (position effects), ecological conditions, and/or linkage-derived genetic load [6]. Linkage-disequilibrium or genetic load could be one of the main causes of fitness depression witnessed in hybrid and backcrossed generations [6]. Until now, the specific effects of linkage-derived genetic load have not been empirically tested under agronomic conditions.

Muller [17] first described genetic load as the total amount of deleterious mutations in the genome of an organism. Since then, genetic load has been studied in the contexts of population and conservation genetics, but not in regards to transgenic plants. Here we define genetic load as the unfavorable consequences of hitchhiking crop or domestication alleles (i.e. crop alleles linked to transgenes) as they become introgressed into a weedy genome [2,6,18-21]. Crops have traditionally been bred and selected for domestication traits, such as lack of seed dormancy, reduced seed dispersal, and non-shattering pods, apical dominance, homogenous fruit ripening, reduced competitive ability, and loss of self-incompatibility [2]. In contrast, weeds have been selected for "weediness" traits that are the counterpoint for the crop traits listed above [22]. Therefore, it follows that the incorporation of crop alleles into weedy genetic backgrounds would be disadvantageous for weedy recipients [2,6,22].

The purpose of this study was two-fold. The first objective was to test the hypothesis that genetic load could have a negative effect on introgression and transgene persistence. Specifically, that hybrid inferiority in *B. rapa × B. napus *populations could be caused by the genetic load of crop alleles that are inadvertently transferred with the transgene into the genome of the weed host. By measuring *Brassica *hybrid and parental productivity (seed yield and biomass), under competitive (grown with a wheat crop) and non-competitive field conditions, the first objective was designed to assess the fitness consequences of genetic load in different competitive environments. These fitness data were then correlated with genetic load, as assessed by number of amplified fragment length polymorphism (AFLP) markers specific to the crop parent (*B. napus*) and to the weed parent (*B. rapa*), to determine whether weed × crop hybrids with more crop genes were less competitive than hybrids containing fewer crop genes. Our hypothesis was that hybrids possessing more *B. napus*-specific AFLP markers would be less fit under both competitive and non-competitive conditions.

The second objective of this study was to analyze the advantages of utilizing a transgenic-mitigation (TM) strategy (engineered genetic load) to reduce the number or frequency of transgenic progeny under field conditions. A number of gene flow prevention models have been proposed in the literature [15,23]. These include: use of buffer zones or barrier crops to block or hinder pollen flow [24], production of male-sterile crop plants to prevent pollen flow [25], insertion of gene-deletor constructs (recombinase system that would excise transgenes from pollen grains) [26], insertion of transgenes in "safe" integration sites or areas that are less likely to be transferred during homologous recombination, thereby inhibiting the transgene from being transferred to subsequent generations [27], insertion of transgenes into the maternally inherited plastid genomes [23,28,29], and TM strategies [16,30-34]. Of these proposed strategies, TM is a focal point of this study.

Our hypotheses was that direct incorporation of engineered genetic load via TM, would be a stronger mitigator of transgene persistence than random transgene-site-mediated genetic load, which can easily undergo trait segregation in surviving offspring. This is a follow-up field study from greenhouse and shade-house experiments that demonstrated that the above TM strategy was effective in limiting seed production and thereby mitigating transgene flow from *B. napus *to *B. rapa *[33,34]. We chose the BC_1_/F_2 _generation since it is the first generation towards introgression in which genetic segregation is expected to vary among transgenic events.

Transgene mitigation is made possible by fusing or linking agronomically beneficial transgenes (i.e. a gene conferring herbicide, insect, or disease-resistance) with transgenes that would decrease the fitness of the weed host, hence inhibiting the persistence of the TM construct. These same weed-mitigating genes are either positive or neutral for the crop [30,35]. The mitigating gene used in this study was the *Δgai *(gibberellic acid insensitive) gene. This gene confers a dwarf phenotype, a principal trait of the Green Revolution. Incorporation of this gene has been shown to increase crop biomass and seed yield, but render weeds less competitive [33,36]. The fitness consequences of this engineered genetic load was also estimated in the present study from *Brassica *hybrid and parental seed yield and above-ground dry biomass measures, under competitive (grown with a wheat crop) and non-competitive field conditions. It was hypothesized that TM would be able to eliminate the persistence of transgenes in progeny and derivative populations, or keep them at exceedingly low frequencies if gene flow and subsequent transgene introgression were to occur.

## Results

### Genetic load study

#### Productivity data: no competition

**Figure 1:**
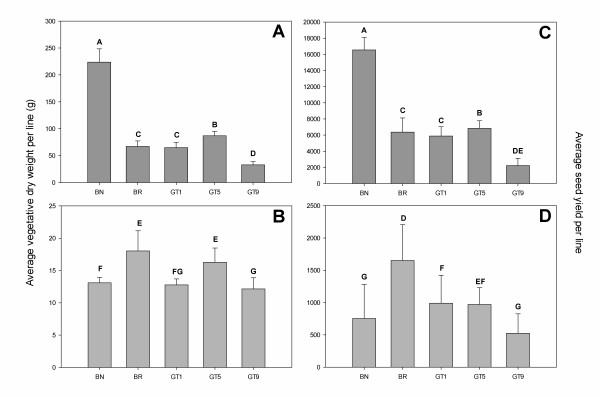
**Genetic Load Study: Productivity**. Average vegetative dry weight and seed yield (2e +4 = 20,000 seeds, 1e + 5 = 100,000 seeds, etc.) of non-transgenic *Brassica napus *(BN), *Brassica rapa *(BR) and transgenic BC_1_/F_2 _hybrid lines (GT1, GT5 and GT9) grown under non-competitive **(A and C) **and competitive field conditions **(B and D)**. Columns with the same letter do not differ statistically (P < 0.0001). Error bars represent ± standard error of the means. Note that different Y-axis scales are used among figure panels.

When lacking competition, from wheat or weeds, non-transgenic *B. napus *out-performed *B. rapa *and the three hybrid lines (Figures 1A and 1C). The GT5 hybrid line had significantly (P < 0.01) higher seed biomass than *B. rapa*. The other hybrid lines, GT1 and GT9, were significantly less fit (P < 0.01) than *B. rapa*. The same pattern was observed for vegetative dry mass. Regardless of treatment or line, there was a strong correlation between biomass and seed yield (r = 0.83, P < 0.0001) (data not shown).

#### Productivity data: competition with wheat

When the two non-transgenic parental lines and three GT hybrid lines were grown in competition with wheat, *B. rapa *produced the most, and *B. napus *and GT9, the fewest seeds (Figure 1D). There were no statistical differences between the GT1 and GT5 hybrid lines for seed production. The GT9 line produced significantly (P < 0.01) fewer seeds than the other hybrid lines and was not significantly different that *B. napus*. However, all of the hybrid lines produced significantly (P < 0.01) fewer seeds than *B. rapa *(Figure 1D) under competitive conditions. Two of the hybrid lines had lower biomass than *B. rapa*, whereas the GT5 hybrid line produced as much biomass as *B. rapa *in competition with wheat (Figure 1B). No statistical differences were observed between non-transgenic *B. napus and *GT1 and GT9 hybrid lines when they were grown in competition with wheat (Figure 1B). The effect of *Brassica *competition on wheat productivity differed among lines, but overall, no significant difference was observed between wheat growth in the absence of *Brassica *competition vs. the presence of *Brassica *competition under the specific conditions of this experiment (data not shown).

#### AFLP marker analysis

The five selective primer sets used [18] resulted in a total of 136 *B. napus-*specific markers. A marker was considered *B. napus-*specific if it was present in the bulked *B. napus *sample and absent in the bulked *B. rapa *sample. The five selective primer sets also resulted in 95 *B. rapa-*specific markers. A marker was considered *B. rapa-*specific if it was present in the bulked *B. rapa *sample and absent in the bulked *B. napus *sample. As the *B. rapa-*specific markers were absent in the *B. napus *crop, they can be considered markers of weediness, the more present, the weedier the hybrid or segregant.

**Figure 2:**
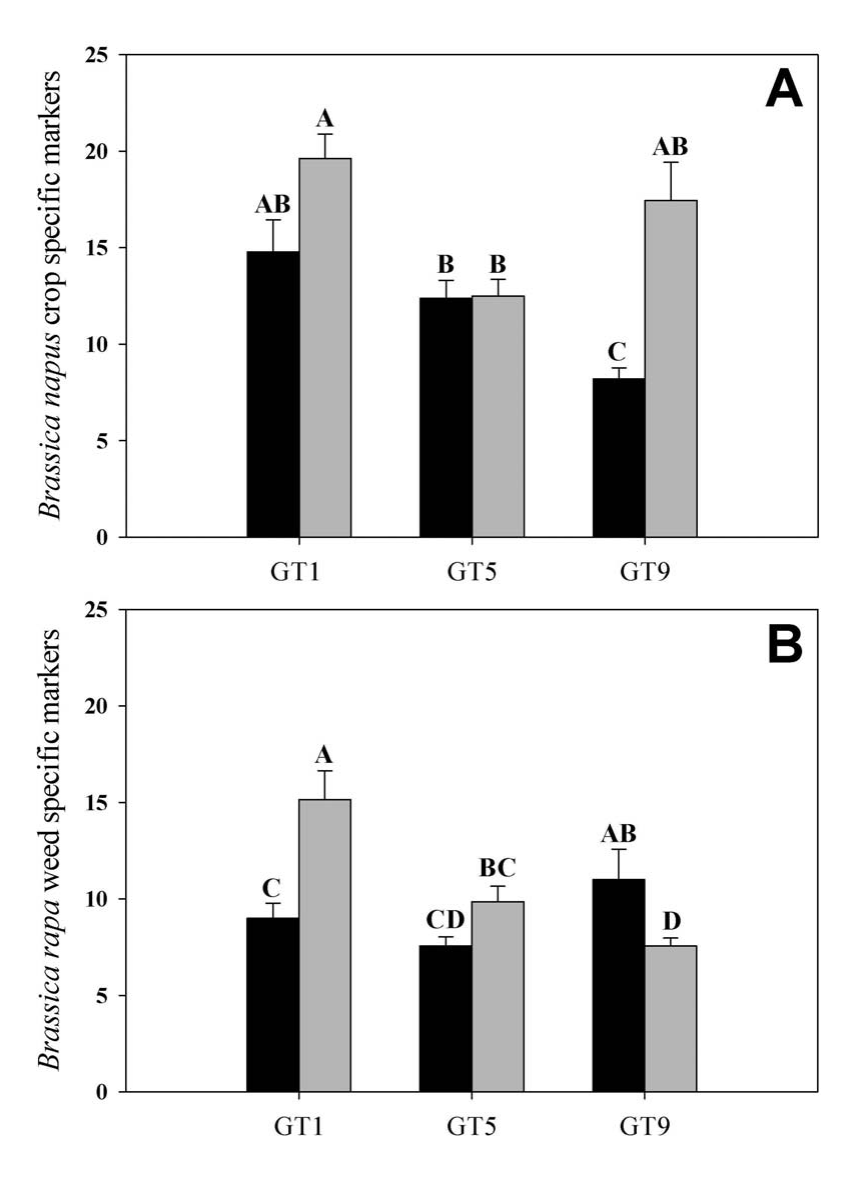
**Genetic Load Study: AFLP Markers**. **(A) **Average number of *Brassica napus *crop-specific- and **(B) **Average number of *Brassica rapa *weed-specific AFLP markers in the transgenic BC_1_/F_2 _hybrid (GT1, GT5, GT9) populations as a result of being grown under non-competitive (no-wheat competition plots) (black columns) and interspecific competitive conditions (lines grown in wheat plots) (gray columns). Columns with the same letter do not differ statistically (P < 0.0001). Error bars represent ± standard error of the means.

The number of *B. napus- *and *B. rapa-*specific AFLP markers differed among the GT hybrids and treatments (competition with wheat vs. no-competition with wheat) (P > 0.0001) (Figure 2). In the absence of interspecific competition, GT9 hybrids had significantly more *B. rapa *markers and significantly fewer *B. napus *markers than the other lines. However, when GT9 hybrids were grown in competition with wheat, GT9 had significantly more *B. napus *markers (actually not different than GT1) and significantly fewer *B. rapa *markers than the other lines in the competition treatment. In contrast, more *B. rapa *markers were measured in the GT1 hybrids grown in competition with wheat than in GT1 hybrids grown under no competition, while no differences were seen in *B. napus *markers for either of the treatments. Thus, GT1 and GT9 and different patterns of response to competition with regards of genomic constitution. In the GT5 line, there were no significant differences between the number or type of marker in either of the growing conditions (Figure 2). Even though significant differences were observed for the markers and lines under different competitive conditions, correlations were only observed between markers and productivity data for the GT1 hybrids (see below).

#### *B. napus *AFLP marker correlation with productivity

**Figure 3:**
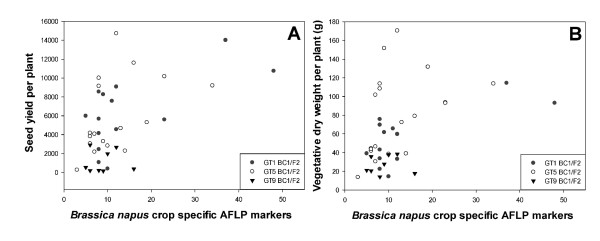
**Genetic Load Study: Productivity and *B. napus*-specific AFLP markers Correlation**. Correlation analysis between **(A) **seed yield (A) (global analysis: r = 0.54, P > 0.0003), **(B) **vegetative dry weight per plant (global analysis: r = 0.44, P > 0.0046), and *B. napus*-specific AFLP markers under non-competitive conditions. Each data point represents an individual plant from the transgenic BC_1_/F_2 _hybrid (GT1, GT5, GT9) populations.

Our hypothesis that hybrids containing more *B. napus *crop alleles or genetic load would be less competitive than weedy species can be conditionally rejected, but only in the case where there is no interspecific competition (Table 1). There was an overall moderate positive correlation between the number of *B. napus-*specific AFLP markers and seed yield and vegetative biomass under non-competitive conditions: for seed yield vs. marker number (r = 0.5, P < 0.0003) (Figure 3A) and vegetative dry biomass vs. marker number (r = 0.4, P < 0.005 (Figure 3B). The statistical significance can be accounted for by GT1 hybrids (Table 1). No correlations were observed for the other events. When the GT hybrids were grown in competition with wheat, there were no significant correlations between the number of *B. napus *crop markers and seed yield, vegetative dry biomass or with wheat vegetative dry biomass (Table 1). In addition, no correlations between *B. napus *markers and wheat vegetative dry biomass existed for any of the hybrid lines (Table 1).

**Table 1:** Genetic load study: correlation analysis between *Brassica napus *and *Brassica rapa *AFLP genomic markers and hybrid productivity.

Correlation results between *Brassica napus *crop-specific AFLP markers and hybrid productivity					
	**No Competition**		**Competition**		

**Line (BC_1_/F_2_)**	**Seed Yield**	**Vegetative Biomass**	**Seed Yield**	**Vegetative Biomass**	**Wheat Biomass**

**GT1**	0.6*	0.7*	-0.2	-0.4	0.2
**GT5**	0.4	0.4	0.3	0.4	-0.1
**GT9**	0.1	-0.01	-0.5	-0.4	0.1

**Global Analysis**	0.5*	0.4*	**0.1**	**0.1**	**0.1**

**Correlation results between *Brassica rapa *weed-specific AFLP markers and hybrid productivity**					

	**No Competition**		**Competition**		

**Line (BC_1_/F_2_)**	**Seed Yield**	**Vegetative Biomass**	**Seed Yield**	**Vegetative Biomass**	**Wheat Biomass**

**GT1**	0.4	0.4	0.8*	-0.2	0.2
**GT5**	0.4	0.4	0.4	-0.1	-0.1
**GT9**	0.3	-0.1	0.3	0.1	0.1

**Global Analysis**	**0.1**	**0.1**	0.7*	**-0.1**	**0.1**

* Denotes significant results (P < 0.01)

The numeric values represent Pearson correlation coefficients (r).

Hybrids were grown in the presence and absence of interspecific competition with wheat.

#### *B. rapa *AFLP marker correlation with productivity

**Figure 4:**
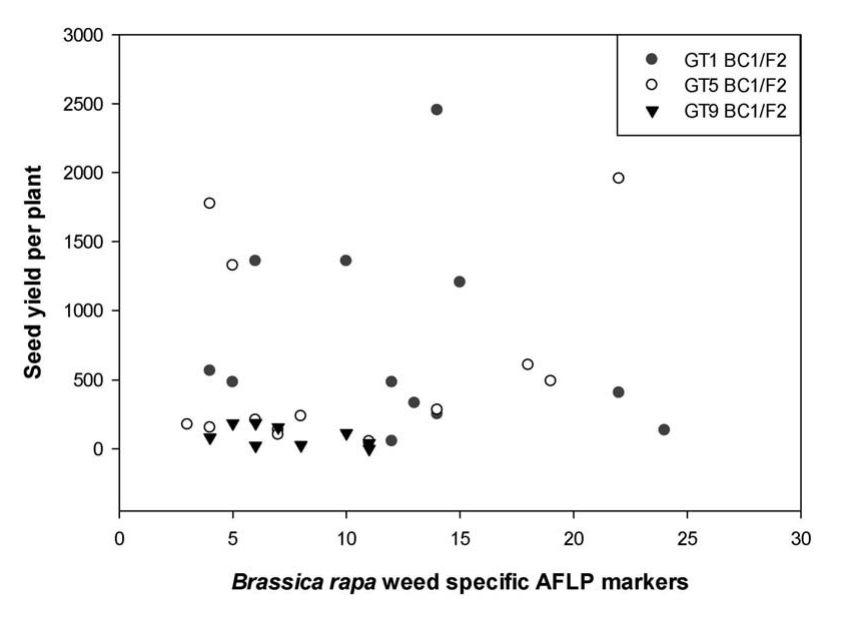
**Genetic Load Study: Productivity and *B. rapa*-specific AFLP markers Correlation**. Correlation analysis between seed yield per plant (global analysis: r = 0.70, P > 0.0001) and *B. rapa*-specific AFLP markers under competitive conditions. Each data point represents an individual plant from the transgenic BC_1_/F_2_hybrid (GT1, GT5, GT9) populations.

There were no correlations between the number of *B. rapa *specific AFLP markers and the productivity of the GT hybrids grown alone, i.e., in the absence of interspecific competition (Table 1). Conversely, under competitive conditions (i.e. when the hybrids were grown amongst wheat), there were strong positive correlations between *B. rapa *weediness markers and seed yield but only for the GT1 hybrids. In the GT1 line, the correlations between *B. rapa *markers under competitive conditions were slightly higher than those for the *B. napus *markers under no competition: seed yield vs. marker (r = 0.8, P < 0.0002) (Figure 4). Overall, the correlations between the number of *B. rapa *markers and seed yield under competitive conditions were significant (r = 0.7, P < 0.0001). No correlations were observed in the GT5 and GT9 hybrid lines with respect to the *B. rapa *markers and seed yield or vegetative dry biomass. In addition, no correlations between *B. rapa *markers and wheat vegetative dry biomass existed for any of the hybrid lines (Table 1).

### Transgenic-mitigation study

#### Productivity data

**Figure 5:**
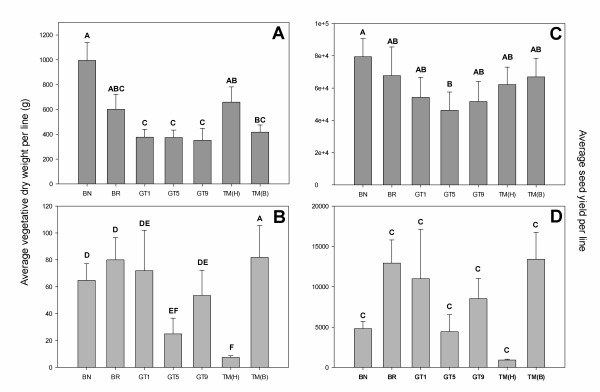
**Transgenic Mitigation Study: Productivity**. Average vegetative dry weight and seed yield (2e +4 = 20,000 seeds, 1e + 5 = 100,000 seeds, etc.) of non-transgenic *Brassica napus *(BN), *Brassica rapa *(BR), homozygous TM [TM(H)] and transgenic BC_1_/F_2 _hybrid populations [GT1, GT5, GT9 and TM(B)] being grown under non-competitive **(A and C) **and competitive field conditions **(B and D)**. Bars with the same letter do not differ statistically (P < 0.0001). Error bars represent ± standard error of the means. Note that different Y-axis scales are used among figure panels.

For the TM study, we analyzed populations of non-transgenic *B. napus *and *B. rapa*, a transgenic homozygous TM [TM(H)] *B. napus *population and transgenic segregating populations of BC_1_/F_2 _hybrids [GT1, GT5, GT9 and TM(B)]. The GT hybrids used in the TM study were the same lines that were analyzed in the genetic load study, whereas the TM(H) and the TM(B) lines were unique the TM study. When grown in competition with wheat, the non-transgenic *B. napus *and *B*. *rapa *plants reached heights of 130-160 cm at plant maturity (5 months after planting) whereas the homozygous TM *B. napus *plants [TM(H)] were only 40-60 cm at plant maturity. At the time of harvest (early June), the siliques on all of the non-transgenic and hybrid plants were completely mature and ready to shatter, while the siliques on the TM(H) plants (under competitive and non-competitive conditions) were still green-to-yellow and maturing. Throughout the field season, it was observed that the TM(H) line had delayed emergence, reduced height, and delayed flowering. However, based on productivity data, when grown alone, the TM(H) populations performed as well as their non-transgenic crop counterpart (*B. napus*), indicating that dwarfing could potentially be utilized in true cropping systems without any yield penalties (Figure 5A and 5C). These results are congruent with those of Al-Ahmad et al. (2006a).

When populations of transgenic and non-transgenic segregants of the GT and TM hybrid lines were grown in the absence of competition with wheat, significant (P < 0.01) differences in biomass and seed yield were observed (Figure 5A and 5C), however, all hybrid lines [GT1, GT5, GT9 and TM(B)] performed similarly to *B. rapa *in the absence of competition. In contrast under competitive conditions, there were no statistical differences observed for vegetative biomass (Figure 5B) or seed yield (Figure 5D) for any of the hybrid lines, but there was a great deal of endogenous variation among data in the competition experiment.

#### Transgene persistence data

After one field season of natural hybridization among individuals of a hybrid line, the progeny from the BC_1_/F_2 _hybrid lines was comprised of BC_2 _and F_3 _individuals. Transgenic segregation ratios in the progeny populations differed among the GT and TM lines (Table 2). The frequencies of transgenic individuals decreased in all GT and TM hybrid progeny populations after one field season. The frequencies of transgenic individuals in these populations differed among lines and under interspecific and intraspecific competitive conditions (P > 0.0001). The proportion of transgenic individuals in the progeny populations of the GT1, GT5, GT9 and TM BC_1_/F_2 _hybrid lines were the same for both non- and competitive conditions (Table 2). However, there was a significant difference observed for the TM(H) line. The progeny population of the TM(H) line grown in competition with wheat had fewer transgenic individuals than that grown in the absence of interspecific competition (Table 2). The green fluorescent marker (GFP) was also detected in the parental (BN and BR) progeny lines indicating the occurrence of gene flow among plots (Table 2). The F_1 _progeny populations for the BN and BR parental lines were not screened on MS medium containing the selecting imazapyr herbicide, Hence, the transgene persistence data reported in Table 2 only applies to gene flow from GT hybrid plots and not TM hybrid or homozygous plots. The decrease in transgenic individuals was greater for the TM hybrid line than the GT lines. The initial frequency of transgenic individuals in the TM hybrid line [TM(B)] was low (22% of the population), and after one field season, the frequency of transgenic individuals dropped further (i.e. less than 1%: 0.03% in competition and 0.07% in no competition) (Table 2).

**Table 2:** Transgenic mitigation study:percent germination and transgenic segregation frequencies under non-competitive and competitive conditions.

	Parent Populations				Progeny Populations: Competition				Progeny Populations: No Competition			
**Line**	**Seeds Plated**	**Germination (%)**	**Transgenic Ratio (%)**	**MS**	**Seeds Sown**	**Germination (%)**	**Transgenic Ratio (%)**	**MS**	**Seeds Sown**	**Germination (%)**	**Transgenic Ratio (%)**	**MS**

**BN**	125	100	0	K	125	93.9	1.4	IJ	125	89.1	2.8	HI
**BR**	125	100	0	K	125	92.2	7.3	G	125	89.8	5.7	GH
**GT1**	125	80.7	55.1	C	125	89.9	38.5	D	125	88.6	36.8	D
**GT5**	125	79.3	63.9	BC	125	88.9	34	D	125	93.3	37.1	D
**GT9**	125	90.7	32.1	DE	125	90.7	10.3	FG	125	89.8	10.4	FG
**TM(H)**	125	86.4	100	A	125	81.6	77.7	ABC	125	82.6	88.3	AB
**TM(B)**	125	87.2	22.4	EF	125	84.3	0.4	JK	125	87.3	0.7	IJK

Percent germination and transgenic frequencies were calculated for the parental (BN: *B. napus*; BR: *B. rapa*; TM(H) and hybrid BC_1_/F_2 _populations (GT1, GT5, GT9, and TM) pre-season and post-harvest. Transgene persistence data reported here only applies to gene flow from GT hybrid plots and not TM hybrid or homozygous plots. Column MS represents transgenic ratio mean separation obtained by Fisher's LSD (P < 0.0001). Data was rank transformed and SAS software was used for data analysis.

## Discussion

There is growing evidence that many interspecific hybrids between crops and weeds, including transgenic hybrids, have inferior performance compared with their weedy parent (reviewed in [37]), although a few exceptions have been reported [13,38]. There are many possible explanations for these observations, most of which focus on the fitness penalties associated with hybridization and backcrossing. A variety of genetic mechanisms can affect hybrid fitness [39]. Heterosis or hybrid vigor could be enhanced by the segregation of additive genetic traits or optimal environmental conditions. In cases where the crop and weedy or wild relative are the same species, there should be fewer genetic barriers to hybrid fitness and transgene persistence [37,40,41]. Unless the trait or transgene is deleterious, as in the case of TM, hybridization and introgression might easily progress, thereby potentially allowing transgene persistence. This is far more likely to occur with related weeds existing and competing in the same agro-ecosystem as the crop than with wild species, residing in non-agricultural areas where a transgenic trait may have little or no value. The performance of new genotypes introduced via transgenesis and subsequent hybridization are likely also subject to genotype × environment (G × E) interactions [3-7,41-43].

When distantly related taxa hybridize, recombination between homologous or homoeologous chromosomes can result in irregular chromosome pairing. The latter can lead to reduced growth and fertility [37], thus decreasing potential for introgression. In addition, the dilution of weed alleles by crop alleles, i.e., genetic load, might also account for slower introgression regardless of environment [2,6]. Our results showed a positive correlation between genetic load (estimated by *B. napus*-specific AFLP marker numbers) and plant productivity in only one of our transgenic events, GT1, a result congruent with a prior study [6]. No correlation was observed for the other transgenic events suggesting a strong transgenic event or genotype effect. The experimental design allowed the detection of a surprising effect--a counter genetic load finding (i.e. genetic load, conferred by crop alleles, was not detrimental to hybrid performance as originally hypothesized). In our experiments, the number of *B. napus *crop-specific AFLP markers was associated with an increase in hybrid performance (as opposed to a decrease in performance) in the GT1 hybrids in the absence of interspecific competition with wheat and weeds. Conversely, in the presence of interspecific competition with wheat, there was a strong correlation between *B. rapa *weed-specific AFLP markers and productivity, again, only for the GT1 line. These results indicate a strong G × E interaction and that inherited crop alleles, introgressed as a result of hybridization, can be preferentially selected to ensure maximum growth potential and productivity under crop-favorable conditions (as seen in the absence of interspecific competition). Conversely, inherited weedy alleles can be preferentially selected under more "weed-like" conditions (i.e. under interspecific competitive conditions) [6]. We anticipate that this will continue to occur as the lines continue to backcross.

This evidence of local and situational adaptation demonstrated in our study as well as other studies indicates that many alleles are beneficial in some but not all environments [40-44]. As sessile organisms, plants must be able to maximize resource acquisition; this can be accomplished with adapted genomes. In agriculture and in our study, environmental genomic adaptation could translate to favoring some crop alleles in crops grown in monocultures where weeds are controlled; weed alleles might be favored in weeds in competitive environments [22]. Dilution in either direction could be maladaptive. Environmental adaptation and trait selection are further demonstrated in a case-study performed on recombinant inbred sunflower lines [40]. Baack et al. [40] showed that crop traits (earlier flowering time, larger stem diameter, and larger flowering disk diameter) were preferentially favored in one site, while weedy traits (smaller ray and seed size) were favored at another location [40]. These studies indicate that, with regards to genetic load and trait selection, environmentally-dependent selection must be taken into consideration for every transgene, species, and situation studied [40,42-44].

### Transgenic event effects and genetic load

Since transgene insertion is random, the flanking host DNA can differ significantly for individual transgenic events; hence the analysis of multiple events in the present study. Zhu et al. [45] examined the transgene segregation ratios for each of our lines using controlled crosses and progeny analysis. The GT1 and GT5 lines did not deviate from expected Mendelian segregation, indicating homeologous recombination of the transgene locus on an A-genome chromosome (the A genome is shared by the two parental taxa, *B napus *AACC- and *B. rapa *AA genomes), and hence the transgene would have a decreased chance of being lost in subsequent backcrossing as a result of genomic incompatibility. Thus, linkage effects could likely cause decreased introgression and genetic load in the GT1 line. Transgenes located on the C-genome of *B. napus *might be subject to greater genomic incompatibility since *B. rapa *does not have the C-genome of *B. napus*. Thus C-genome-localized transgenic events could be an important investigative avenue for decreasing introgression [2,27], although recent research found that homeologous recombination occurs at the same rate as homologous recombination in the *B. rapa × B. napus *system [29,46], suggesting that "safe-integration" sites in *B. napus *are unlikely [46].

### Engineered vs. randon transgenic event genetic load

TM *B. napus *plants containing the dwarfing gene *Δgai *were previously analyzed under shade-house conditions [31-34]. Homozygous TM dwarf *B. napus *plants [TM(H)] grown alone (in the absence of competition) had a much higher seed yield (P < 0.01), and double the shoot and root biomass compared to non-transgenic counterparts (P ≤ 0.01). The TM(H) line also produced more leaves than tall non-transgenic plants when grown alone at 2.5-cm spacing (P ≤ 0.05) and 10-cm spacing (P ≤ 0.05). However, when grown in competition with tall non-transgenic cohorts, these same plants were exceedingly unfit [33]. The reproductive fitness of TM(H) plants, when grown in competition, was less than 12%, and the harvest index (grain-to-straw ratio) was less than 40% of that of the non-transgenic *B. napus *competitors. When grown in the absence of competition, the TM(H) line produced the greatest amount of seeds per plant [33]. From these studies, Al-Ahmad et al. [33] concluded that the Δ*gai *gene greatly enhanced seed and biomass yield in a weed-free transgenic crop. However, if dwarfed plants emerged as volunteer weeds and competed with non-transgenic cohorts (and presumably other species), dwarfed plants would be eliminated from poor competitive ability, especially if selective herbicides were not used.

Our field results were congruent with the previous TM research [31-35]. When the homozygous TM line [TM(H)] was grown under agronomic non-competitive conditions, it had equivalent performance as the non-transgenic *B. napus *line with regards to seed yield and biomass. However, under competitive conditions, the TM(H) line produced the least seed and biomass and, hence, performed the poorest of all the lines that were analyzed. In terms of transgene persistence, the transgenic progeny from the backcrossed TM(B) line approached a 50- to 85-fold reduction, under competitive and non-competitive conditions, compared with the 3-fold reduction in transgene persistence with the non-TM (GT) lines. It must be noted that the final portion of the transgene persistence studies; i.e., germination of seed collected from field grown plants were carried out under optimal growth chamber conditions. This environment did not take into consideration the effects of over-wintering survivorship, dormancy issues or seedling dynamics. Consequently, when environmental factors are considered, one could potentially expect a decrease in performance (germination rates, etc) under realistic field conditions. Hence, *ex situ *analysis of transgene persistence could appear higher than it actually would be in the field [6]. Regardless, these data do indicate that TM constructs were effective in severely limiting the impact of gene flow from transgenic crops to their wild relatives and transgene persistence. The TM approach could further be enhanced by stacking other weed mitigating transgenes, such as those that prevent seedpods from shattering or those that prevent secondary dormancy. In addition to making plants shorter the dwarfing gene could also confer pleiotropic effects such as altered flowering traits. These could conceivably also have G × E interactions and effect fitness [42]. Depending on the trait, the pleiotropic effect could increase or decrease fitness. In this particular instance, dwarfed plants were very late flowering and also had delayed seed maturation and decreased germination. In this case, the pleiotropic effects would almost certainly decrease introgression.

Some researchers point out that dwarfing in TM is a trait that could foreseeably be selected against in the hemizygous state; e.g., in semi-dwarfed plants [45,46]. Consequently, the dwarfing allele might not be expressed as highly in first-generation hybrids because of the presence of the dominant *GAI *allele from the weedy parent genome. Therefore, hybrids containing the TM construct might not have sufficient dwarfing to be selected against in competition, yet harbor the fitness-enhancing gene from the TM construct. In subsequent generations, the deleterious allele would only be expressed in homozygous individuals, which would strongly reduce its capability to decrease fitness. Moreover, if the hybrids are fertile (as is the case for *Brassica *TM hybrids) [34], this strategy would not prevent them acting as a genetic bridge and pollinating the wild parent [37].

Reuter et al. [47] challenged the theory that dwarfed volunteers or transgenic escapees would be less competitive in feral environments. They noted that feral non-transgenic *B. napus *populations, growing in rural and urban area in northern Germany, were, on average, 41% shorter than cultivated non-transgenic *B. napus*. They attributed this height difference to phenotypic adaptation to local environments. Reuter et al. [47] concluded that, under certain environmental and ecological conditions, the proposed mitigation approach (dwarfing) could actually increase escape and persistence of transgenic varieties rather than reducing them, but their study did not consider genotype differences, local adaptation, and environmental effects such as differences in nutrient availability between agronomic and feral conditions. Our transgene persistence data indicate that even if the dwarfing trait is in the semi-dominant state, it is still effective in limiting transgene persistence. Our results indicate that when dwarfing is utilized for a containment strategy, the plants are not able to compete as well and hence persist in subsequent generations. These observations hold true regardless of whether the plants were grown in the presence or absence of interspecific competition. Hence, under the conditions and situations analyzed in this study, hybrids containing TM constructs were not effective weeds.

Different species will likely require various mitigators, and possible also for different environments [16]. Anti-shattering genes will be appropriate for many row crops that have seed shattering weedy relatives, and anti-secondary dormancy genes would be appropriate where weeds possess seed-bank longevity as part of their survival mechanisms. Anti-bolting genes (e.g. anti-kaurene oxidase preventing gibberellic acid biosynthesis) would be appropriate mitigators for biennial crops (carrots, beets) or storage crops (onions, radishes, cabbages) that have weedy relatives. Sterility genes are appropriate for vegetatively propagated species (potatoes, poplars). The waxy transgene in maize would be a good mitigator for pharmaceutical genes expressed in maize embryos, as seeds from self or cross pollination would be shrunken, and unable to survive over-winter in most soils [16]. Thus, to choose an appropriate mitigator for each crop and environment, the researcher must seek out genes that are positive or neutral for the crop in that environment and would be detrimental to the related weedy or wild species.

## Conclusion

There is a paradox regarding the apparent absence of introgression in species where gene flow is expected on the basis of sexual compatibility data. It might be that genetic load of endogenous genes on crop chromosomes moderates introgression, and this same effect could be extended and applied to transgene containment. Perhaps we do not observe extensive transgene persistence in weedy relatives, i.e., only one introgressed transgenic weedy *B. rapa *plant has been observed in the field despite the extensive commercial release of transgenic *B. napus *[20], because of genetic load conferred by the lack of homeologous recombination or hitchhiking of crop alleles. If additional genetic load is imposed using TM, it could dramatically decrease introgression and transgene persistence to an even lower level.

## Methods

### Parental species

*Brassica napus *(canola, oilseed rape, OSR) is grown worldwide as an oilseed-producing crop and, after soybean, ranks second in edible oil production. *B. napus *is an allotetraploid (AACC genome, 2n = 38) and evolved through hybridization and polyploidization between the two diploid species *B. rapa *(2n = 20, AA) and *B. oleracea *(2n = 18, CC) [29,48]. *B. napus *is an excellent model crop for the study of genetic load because it can hybridize with closely related weedy species such as *B. rapa *(field mustard, wild turnip, birdseed rape) [2,4,5,18,19,21,23,27,49-55], *Hirschfeldia incana, B.a juncea *and, to a lesser degree, with more distant relatives such as *Raphanus raphanistrum *(wild radish) [[19,56], reviewed in [57]]. However, hybridizations between *B. napus *and *B. rapa *are the most common and often produce the most viable offspring [57].

*B. rapa*, is a common weed that can be found in and around many areas of *B. napus *cultivation. Consequently, *B. rapa *can be a nuisance to farmers because it can compete with and hence reduce the yield of crops such as *B. napus *and wheat [4,5,18,19,27,52-55,58]. The presence of weedy *B. rapa *is a major problem in parts of Canada, the US, and in the UK where OSR is grown as a staple crop for oil production [19].

### Plants

#### Genetic load study

*B. napus *cv "Westar' transgenic events were from Halfhill et al. [4]. They contained constitutively-expressed green fluorescent protein (GFP), *mGFP5er*, and synthetic *Bacillus thuringiensis *(synthetic *Bt cry1Ac*) under the control of separate 35S promoters and contained a kanamycin resistance cassette. These lines were labeled as "GT" lines because they contain *GFP *and *Bt *transgenes. Of the nine independent transgenic events (GT1-9) that were produced by Halfhill et al. [4], we used three (GT1, GT5, and GT9) in both experiments in this study. Sister-hybrid lines, which contain the same transgene construct but in different insertion loci [6,37,39], were produced by hybridization with a single *B. rapa *accession (acc. 2974) from Quebec, Canada [6] to produce BC_1_/F_2 _populations (see below). Non-transgenic *B. rapa *(acc. 2974) and *B. napus *(cv Westar) parental lines were used as controls in the genetic load study (Table 3).

**Table 3:** Plant germplasm.

Line	Experiments	Transgenic?	Generation	Parental Lines	Purpose
*Brassica napus *(BN)	Both	**no**	n/a	n/a	crop: parental control
*Brassica rapa *(BR)	Both	**no**	n/a	n/a	weed: parental control
GT1 hybrid	Both	**yes**: *mGFP5er *and *Bt cry1Ac*	mixed: BC_1_/F_2_	BR and transgenic BN	to study event-specific genetic load
GT5 hybrid	Both	**yes**: *mGFP5er *and *Bt cry1Ac*	mixed: BC_1_/F_2_	BR and transgenic BN	to study event-specific genetic load
GT9 hybrid	Both	**yes**: *mGFP5er *and *Bt cry1Ac*	mixed: BC_1_/F_2_	BR and transgenic BN	to study event-specific genetic load
TM homozygous [TM(H)]	TM only	**yes**: Δ*gai *and *ahas*	T_2_	BN	TM parental control
TM backcross [TM(B)]	TM only	**yes**: Δ*gai *and *ahas*	mixed: BC_1_/F_2_	BR and transgenic BN	To study transgene persistence in a backcrossed population

Summary of line designations, generations and parental lines for the seven plant germplasm types utilized in this study. The transgene nomenclature is as follows: *mGFP5er *(gene coding for green fluorescent protein); *Bt cry1Ac *(*Bacillus thuringiensis *insecticidal toxin gene); Δ*gai *(gibberellic acid insensitive gene); *ahas *(acetolactate synthase-herbicide resistance gene).

#### Transgenic mitigation study

All of the above-mentioned *Brassica *lines (*B. napus, B. rapa *and GT1, GT5 and GT9 BC_1_/F_2 _populations) were utilized along with two additional plant types: TM J9 T_2 _*B. napus *[labeled as TM(H) throughout this manuscript; the "H" represents homozygous and TM BC_1_/F_2 _mixed hybrid population (labeled as TM(B) throughout this manuscript, the "B" represents backcross). The transgenic TM(H) event, i.e. *B. napus *cv. Westar transformed with a transgene-mitigating (TM) construct, was from Al-Ahmad et al. [33] and served as the control in the present study in comparisons with a TM BC_1_/F_2 _[TM(B)] mixed hybrid population obtained using the *B. rapa *accession 2974 described above (Table 3). For this study, all lines were grown under intraspecific (no-competition with wheat, competition within a species) and interspecific (grown amongst wheat, competition with a different species) competition conditions in order to assess the effect of competition on transgene persistence. The TM plants contain the pPZP212-*ahas*^R^-*Δgai*-1 tandem construct that confers ALS (acetolactate synthase)-herbicide resistance and dwarfing. The mitigation gene (*Δgai*) used in this study was insensitive to the effects of endogenous and applied GA [31,59,60]. The plants were also kanamycin resistant. The *ahas *and *Δgai *genes were tightly linked to each other in the same orientation with a 15-base pair linker sequence [31].

The TM J9 event [33] was selected for our study because of its high productivity compared to three other TM transformants and because it outperformed its non-transgenic *B. napus *counterparts in both greenhouse and shade-house experiments leading the authors to conclude that this line could potentially be used in future field experiments without any yield penalties [33].

### Experimental hybrid populations

In order to investigate variation in genetic load resulting from different transgenic events, field studies were performed on four genotypically-diverse *B. napus × B. rapa *hybrid populations. These populations were composed of a mixture of BC_1 _and F_2 _individuals. This diverse population type was chosen because it mimics the type of volunteer population that might be found under actual field conditions. Previous research has also shown that Mendelian segregation patterns and the number of crop markers begin to differ among transgenic events in these generations [6,45]. Consequently, this population would provide the most power to discriminate genetic load effects among the events. It would also be the generation pool (i.e. post-F_1_) that would yield the highest degree of crop-marker variability [6,45].

Mixed BC_1_/F_2 _populations were produced in the greenhouse. Homozygous GT transgenic *B. napus *lines and the TM(H) *B. napus *line were hand-crossed with non-transgenic *B. rapa *to produce F_1 _hybrid lines for each transgenic event. Transgenic F_1 _hybrids were confirmed for GFP using a hand-held long wave UV light, after which, the F_1 _hybrids were hand-crossed to *Brassica rapa *and crossed amongst themselves, in order to produce mixed BC_1_/F_2 _populations for each event.

The TM hybrids lack a visual marker and were screened on MS [61] medium containing a discriminatory dose of kanamycin (260 μM) and imazapyr (0.5 μM) after being surface sterilized [33]. After screening, the F_1 _TM plants were transplanted in the greenhouse and then crossed in the same manner as the GT hybrids (described above). The parental and progeny lines were screened under laboratory conditions. The parental seed stocks were screened prior to being sown in the field and the progeny seed stocks were screened post harvest (i.e. transgene persistence data, see below).

### Experimental design and data analysis

Both studies were performed at the Lang Rigdon Research Farm in Tifton, GA, USA (31°27'N 83°30'W) from October 2007-June 2008. In order to characterize the weediness potential of BC_1_/F_2 _hybrids and their non-transgenic parental lines, individual *Brassica *plants or populations were grown in conjunction with a fall planted wheat crop (*Triticum aestivum*, AGS 2000). The hybrids and parental lines were also grown in the absence of interspecific competition to assess their maximum growth and productivity potential. Soil sampling and analysis was performed on the field prior to planting. There were no soil nutrient differences found throughout the field (data not shown). Optimal agronomic practices were followed including fertilizer application, over-head irrigation, and weeding. N-P-K fertilization was applied at levels recommended to adjust fertility for agronomic wheat production. A 2 m drill was used to plant the wheat at a seeding rate of 90 kg per hectare. In the absence of insecticide treatments, plants were subjected to ambient herbivory pressure.

A completely randomized split-plot design with replication was utilized for both portions of this study. Statistical analysis included whole plot treatment and sub-plot interactions. Plant productivity data was analyzed using analysis of variance (ANOVA) using SAS version 9.2 (SAS Institute Inc, Cary, NC, USA). Rank transformation was implemented when the data did not meet equal variance or normality assumptions [62].

In the genetic load study, two-week old *Brassica *plants were transplanted into the field site two weeks after the wheat was planted (*Brassica *plants were started in the greenhouse at the same time that the wheat was planted in the field). Prior to transplanting, GFP confirmation was performed using a hand-held long wave UV light. The field site contained twenty plots (ten wheat and ten no-wheat plots), fourteen sub-plots within each plot (two replicates for each parental line, three replicates for each hybrid line and one wheat only or blank plot) totaling 280 sub-plots. Sub-plots (lines) and each treatment (wheat: competition/no-competition) were randomized using SAS version 9.2.

In the transgenic mitigation study, all of the above conditions apply except that lines were sown in the field at the time that the wheat was planted instead of being transplanted. Instead of one plant per sub-plot, twenty-five seeds from each line/population were sown (hand-scattered) into 1 × 2 m sub-plots. Populations were sown instead of transplanted in order to mimic a volunteer population emerging as weeds in a wheat crop. The field site contained 10 plots (5 wheat and 5 no-wheat plots), 7 sub-plots within each plot (for each of the lines), totaling 70 measurable sub-plots.

At plant maturity (mid-June), *Brassica *(single plant for the genetic load study or populations for the transgenic mitigation study) above-ground vegetative biomass was hand-harvested, dried and recorded. Plant dry weight and seed production were used to estimate total productivity. Wheat, located within a half-meter radius surrounding the *Brassica *plants, was harvested to measure the effects of competition on *Brassica *and wheat productivity.

### Genetic load study: AFLP markers

AFLP analysis was used to estimate the number of specific alleles from either *B. napus *or *B. rapa *in hybrid plants. AFLP analysis was performed as described in Halfhill et al. [18] and Vos et al. [63] with minor modifications. Of the two *EcoRI *+ three primers utilized in Halfhill et al. [18], only the E + AAG selective primer was used in our study. Selective amplification products were analyzed utilizing the CEQ 8000 GenomeLab system (Beckman Coulter, Fullerton, CA, USA). Results were scored utilizing the CEQ AFLP Dominant Scoring Software (bin width: 0.85). *B. napus*-specific markers (i.e., those present within the bulked *B. napus *sample and absent in the bulked *B. rapa *sample were selected and scored accordingly, as well as the reverse case for *B. rapa*-specific markers. Since AFLPs are dominant markers, DNA samples from the parental lines were bulked for analysis for several reasons: (1) to acquire a set of parent-specific markers, (2) to increase discriminatory power, and (3) to eliminate the possibility of false positive marker amplification. The DNA from individuals within the three GT hybrid populations was analyzed by plant. The number of plants sampled per line differed because of tissue availability [18,20]. Differences in the total amount of *B. napus *and *B. rapa *AFLP markers was analyzed per line and for each treatment by analysis of variance (ANOVA) using SAS version 9.2. Correlations, between AFLP markers and plant productivity data, were analyzed in SAS using the PROC CORR program. SAS macros used in this analysis were kindly provided by Dr. Arnold M. Saxton from the University of Tennessee, Knoxville, and can be accessed on the following website http://animalscience.ag.utk.edu/FacultyStaff/ArnoldSaxton.html

### Transgenic mitigation study: transgene persistence data

At plant maturity, all of the plants within an individual plot (representing separate segregating GT and TM hybrid populations as well as TM and non-transgenic parental lines) were harvested. Vegetative dry mass and seed yield data were collected (see above). For the GT hybrid and non-transgenic parental lines, a total of 125 seeds (5 replicates of 25) were plated on moist filter paper and placed in a growth chamber (16 h days, 24°C, and 60 μmol/m^2 ^s). After a week, the GT hybrid and parental lines were screened for GFP (green florescent protein) using a hand-held long wave UV light. TM lines (hybrids and homozygous parental line) were screened on MS containing kanamycin and imazapyr as above. The germination frequency was calculated by dividing the number of seeds that germinated by the number of seeds (25 seeds) that were plated. The transgene persistence frequency was then calculated by dividing the number of transgenic individuals by the number of individual plants that germinated.

## Authors' contributions

**CWR**: Performed all field and lab-based experiments and data analysis described in this study and drafted the document. **RJM**: Assisted in the design and field experiment portion of this study. **HSM and MRR**: Assisted in the field experiment portion of this study as well as data collection and statistical analysis. **MDH**: Co-conceived of the genetic load portion of this study, and participated in its design and coordination. **PLR**: Participated in the design and coordination of field experiments, assisted in field experiments, field maintenance and data collection. **SIW**: Co-conceived of the study and helped to revise the manuscript. **HA**: Developed the TM construct and plants used in this study. Also assisted in editing the manuscript. **JG**: Conceived of the TM portion of this study, and participated in its design and coordination and helped to revise the manuscript. **CNS**: PI, conceived of the genetic load portion of this study and participated in its design and coordinated the collaborations that made this study possible; revised the manuscript. **All authors read and consented to the final version of the paper**.
